# The HER2-low revolution in gynecological oncology: could it be a realistic emerging challenge?

**DOI:** 10.1007/s00428-023-03672-w

**Published:** 2023-10-24

**Authors:** Angela Santoro, Belen-Padial Urtueta, Gian Franco Zannoni

**Affiliations:** 1 General Pathology Unit, Department of Woman and Child’s Health and Public Health Sciences, Fondazione Policlinico Universitario Agostino Gemelli IRCCS, 00168 Rome, Italy; 2 Pathology Institute, Catholic University of Sacred Heart, 00168 Rome, Italy

**Keywords:** Low HER2, Gynaecological cancers, Targeted therapy

## Editorial

Ovarian clear cell carcinoma (OCCC) is a high-grade adenocarcinoma, often endometriosis related, generally diagnosed in younger women and at earlier stages than those with high-grade serous histology [1, 2]. Prognosis at an early stage is favorable, while advanced or recurrent diseases experience a poor outcome. Response to traditional surgical and radio-chemotherapeutical protocols is poorer due to an intrinsic chemoresistance. Adjuvant chemotherapy is recommended for OCCC stage IC2–3 patients by the ESMO/ESGO guidelines [3] and for all OCCC patients regardless of their substage by the NCCN [4]. This discrepancy underlines the uncertainty of the adjuvant chemotherapy role in stage I OCCC patients undergoing complete surgical staging.

Heterogeneous genomic alterations are being identified in OCCCs, in which ARID1A (62%) and PIK3CA (51%) are the most frequently mutated pathways, commonly coexisting [1, 5, 6].

Using the molecular-based approach designed for endometrial carcinoma, most OCCCs fall into the CNL molecular subgroup (higher frequency of NSMP and lower frequency of MMRd signature) [7]. POLEmut and MMRd OCCCs were associated with an excellent prognosis, while the p53abn group was related to advanced FIGO stage, nodal metastases, and a poorer prognosis [7].

Considering the presence of MMRd in approximately 15% of OCCCs, different clinical trials have investigated the role of therapeutical regimes based on immune checkpoint inhibitors in this histotype. Due to the low chemotherapeutical response rate, testing for BRCA1/2 gene status may be suggested also in OCCC patients. However, these mutations are only present in 6% of the cases, meaning only a low subset of patients could benefit from the PARP inhibitors use [1].

Novel strategies focused on targeted therapies, including biological agents, could constitute the most promising treatment options, but they are still in development.

A possible target for therapy is that one represented by the human epidermal growth factor receptor 2 (HER2/ErbB2), well-known for its clinical and therapeutical significance in HER2 + breast (15–30%) and gastroesophageal cancers (7–34%), by conferring them a worse biological behavior, but also a therapeutical chance. In ovarian cancer, HER2 positivity varies considerably (8–66%) and specific data regarding the clear cell histotype is limited, with 14–67% HER2 positivity being reported in small patient cohorts (often < 10); no clinical data of antiHER2 therapy in OCCC are available and results of the antiHER2 therapy benefit are inconsistent [8].

Currently, there are a lot of practical pathological issues regarding HER2 assessment in endometrial.

and ovarian cancer:

- Non-standardized HER2 testing in gynecological oncology
- Wide study variability of HER2 positivity definition by immunohistochemistry (IHC) and in situ hybridization (ISH)
- Different applied criteria/scoring systems, using either IHC or ISH and often not both
- Not clearly established concordance between HER2 antibodies
- Poorly defined differences in the antibodies’ performance
- Frequently described IHC/ISH discordance in gynecological cancers, with higher overexpression than amplification rates and lower ISH amplification ratios, generally reflecting aneuploidy or polysomy in carcinomas
- Undefined optimal HER2 testing algorithm (IHC as primary test vs. ISH as reflex test), depending on the correlation between test type, therapeutic response, and clinical outcome
- Lacking data regarding the heterogeneity of HER2 status and the optimal testing timing (primary or metastasis)

In this complex scenario, the paper of Michaela Kendall Bártů et al. [9] represents a thorough analysis of HER2 status in a stringently classified large clinical cohort of OCCCs (*n* = 118).

Authors have characterized the HER2 status using IHC on TMA and fluorescent ISH (FISH). HER2 amplification/overexpression was found in 6/118 OCCCs (5%). The observed frequency of HER2 overexpression/amplification confirms that antiHER2-targeted therapy could be of value in a subset of OCCC patients, especially in cases of metastatic and/or recurrent tumors.

The author additionally defined 28/118 (24%) cases as HER2-low; these results could be of potentially therapeutic value, given the recent discovery in breast cancer (BC) setting that patients with HER2-low cancer could also benefit from anti-HER2 therapy.

HER2-low BC is a recently characterized subtype of HER2-negative BC with an IHC score of 1 + or 2 + /ISH negative phenotype that has led to reconsider the traditional binary classification of HER2 status according to which only patients with HER2 + BC were thought to benefit from antiHER2 therapies. Trastuzumab deruxtecan has recently been approved by the U.S. Food and Drug Administration for the treatment of patients with HER2-low metastatic BC based on the results of the DESTINY-Breast04 phase III trial [10]. Recent clinical studies have shown that novel antibody–drug conjugates (ADCs) aimed against HER2 offer considerable therapeutic advantages in the treatment of this malignancy [10]. In this way, treatment armamentarium for both triple-negative and hormone receptor–positive BCs exhibiting HER2-low expression are thus rapidly evolving.

This is the first work evaluating the HER2 status of OCCC in the context of the novel paradigm of HER2-zero and HER2-low status. Although the relationship between HER2 expression (HER2-zero vs. HER2-low) and survival lost its significance in the multivariate analysis, suggesting that HER2 status is not an independent prognostic factor; it may be an important therapeutic target for a selected subset of OCCC patients also with HER2-low phenotypes.

Since there are presently no clinical trials for the use of drug conjugates for OCCC, it might be desirable to experiment new therapeutical strategies for HER2-low phenotype in order to obtain a considerable improvement in the overall survival of such patients, similarly to breast and gastric cancers, for which the recent evolving issue of HER2 landscape is of increasing clinical impact.

In this scenario, the pathologic testing and the standardized scoring of tumoral HER2 protein expression and gene amplification may play a crucial role in patient selection for tailored targeted anti-HER2 therapy.

Considering the emerging need to optimize strategies for the HER2 status identification through the development of more sensitive and reproducible HER2 assays, future studies are necessary to resolve different practical pathological issues, including the accurate pathological definition, the intratumoral heterogeneity and its clinical impact, and the specific correlation to therapeutic response of the HER2-zero, low and high positivity.
